# Crystal Structures of a Stabilized β_1_-Adrenoceptor Bound to the Biased Agonists Bucindolol and Carvedilol

**DOI:** 10.1016/j.str.2012.03.014

**Published:** 2012-05-09

**Authors:** Tony Warne, Patricia C. Edwards, Andrew G.W. Leslie, Christopher G. Tate

**Affiliations:** 1 MRC Laboratory of Molecular Biology, Hills Road, Cambridge CB2 0QH, UK

## Abstract

The β_1_-adrenoceptor (β_1_AR) is the site of action of beta blockers used in the treatment of cardiac-related illnesses. Two beta blockers, carvedilol and bucindolol, show distinctive activities compared to other beta blockers and have been proposed as treatments tailored to the Arg/Gly389^8.56^ polymorphism of the human β_1_AR. Both carvedilol and bucindolol are classified as biased agonists, because they stimulate G protein-independent signaling, while acting as either inverse or partial agonists of the G protein pathway. We have determined the crystal structures of a thermostabilized avian β_1_AR mutant bound to bucindolol and to carvedilol at 3.2 and 2.3 Å resolution, respectively. In comparison to other beta blockers, bucindolol and carvedilol interact with additional residues, in extracellular loop 2 and transmembrane helix 7, which may promote G protein-independent signaling. The structures also suggest that there may be a structural explanation for the pharmacological differences arising from the Arg/Gly389^8.56^ polymorphism.

## Graphical abstract

**Graphical Abstract:**

**Figure:**
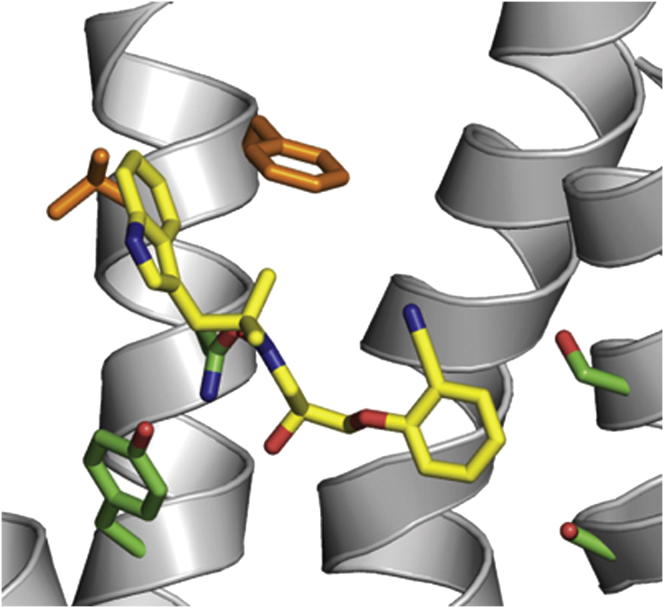

**Highlights:**

► Structures of β1AR bound to the biased agonists bucindololol and carvedilol ► The biased agonists form unique contacts with β1AR not seen with other antagonists ► The structures explain the pharmacological differences in the Arg389Gly polymorphism

## Introduction

The seven-transmembrane-helix receptors (7TMRs) comprise a large and diverse family of cell-surface receptors that on agonist binding can bind and activate a G protein and subsequently initiate diverse signaling events. Some ligands also stimulate G protein-independent pathways (Pierce et al., 2002); unless otherwise stated, the terms “agonist” and “antagonist,” etc. refer to the G protein-coupled pathway (see Box 1 for pharmacological definitions). The 7TMRs are the largest single class of drug target (Wise et al., 2002), and the β-adrenoceptors (βARs) are the targets of beta blockers (antagonists of the G protein-coupled pathway) in the treatment of heart failure, as well as agonists used in asthma therapies. Recent structures of both the β_1_-adrenoceptor (β_1_AR) and the β_2_-adrenoceptor (β_2_AR) have led to an understanding of the molecular characteristics that define an agonist compared to an inverse agonist and how an agonist promotes the activation of a G protein (Cherezov et al., 2007; Hanson et al., 2008; Moukhametzianov et al., 2011; Wacker et al., 2010; Warne et al., 2008, 2011). The structures of the inactive states (R) of βARs when bound to antagonists are very similar, although there are some differences in the region of the “ionic lock” depending on whether the salt bridge between Arg^3.50^ and Glu^6.30^ is present or not (Moukhametzianov et al., 2011) (superscripts refer to the Ballesteros Weinstein nomenclature (Ballesteros and Weinstein, 1995)). When full agonists bind to the R state, the most significant effects are the contraction of the binding pocket and the change in rotamer conformation of Ser^5.46^, implying that these are a prerequisite to the attainment of the activated state (R^∗^) that can couple to G proteins (Warne et al., 2011). In contrast to agonists, partial agonists apparently do not form a hydrogen bond to Ser^5.46^, which explains their decreased efficacy (Warne et al., 2011). The structure of β_2_AR bound to either a G protein mimetic or Gs shows that the agonists bind to R^∗^ in the same manner as to the R state and that the intracellular regions of helices 5 and 6 move by 10-14 Å upon activation (Rasmussen et al., 2011a; Rasmussen et al., 2011b). The challenge over the coming years is to relate the structures of the βARs bound to various ligands and the pharmacological effects of drugs targeting βARs in humans, particularly where the ligands also stimulate G protein-independent pathways, such as through interactions with arrestin.

In the classical view of 7TMR-ligand interactions, agonists bind to receptors and stimulate the activity of specific G proteins to varying degrees, whereas inverse agonists block the effects of agonist activation and also inhibit constitutive signaling. In this view, sometimes termed linear efficacy, the signaling processes associated with a drug's activity are in proportion to its efficacy and therefore its position on the scale of effects ranging from that of a full to an inverse agonist of a G protein, as was shown in early studies on the β_2_AR (Benovic et al., 1988). It is now clear that this is an oversimplification, and the more recent concept of biased agonist function has evolved in response to a body of evidence that shows that 7TMRs can interact directly with other signaling partners, for example, arrestin. These alternative G protein-independent signaling pathways can be selectively stimulated alongside G protein activation, and a ligand's efficacy can be “biased” more or less to different pathways (Rajagopal et al., 2010; Violin and Lefkowitz, 2007). In addition to the implied requirement for distinct conformational states to explain these receptor activities (Kenakin, 2001), there is also a growing realization that existing drugs that target 7TMRs may have more complex effects than first realized, and that the concept of ligand bias may therefore allow the development of more effective therapies (Whalen et al., 2011).

**Figure 1:**
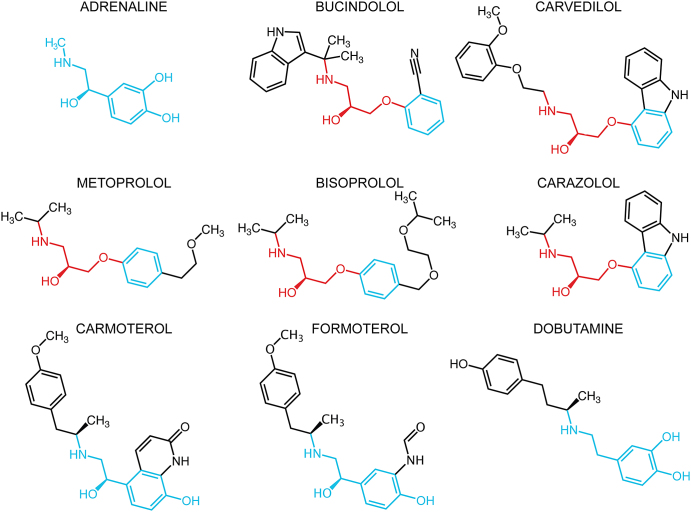
Structures of βAR Ligands Discussed in the Main Text Highlighted regions of the ligands are either conserved with the agonist adrenaline (blue) or are conserved among antagonists (red).

Carvedilol and bucindolol are both beta blockers (antagonists) that target β_1_AR (see Figure 1 for the structures of ligands) and can also bind to β_2_AR. However, both bucindolol and carvedilol have also been shown to stimulate non-G protein-coupled pathways of βARs (i.e., they are biased agonists). With the human β_1_AR, bucindolol has been found to act as a partial agonist of the G protein-signaling pathway as well as an agonist of the mitogen-activated protein kinase (MAPK) pathway through a G protein-independent mechanism (Galandrin et al., 2008). This suggests that bucindolol can induce a signaling conformation of β_1_AR that is distinct from both conventional (G protein) antagonist-bound and agonist-bound states (Galandrin et al., 2008). All conventional βAR agonists can enhance arrestin-mediated signaling as a functional consequence of their activation of the G protein-signaling pathway (Drake et al., 2008; Wisler et al., 2007). However, carvedilol is an inverse agonist of G protein signaling, but it stimulates arrestin-mediated signaling pathways of both human β_2_AR and mouse β_1_AR independently of G protein-mediated signaling (Kim et al., 2008; Wisler et al., 2007). Because of these unique properties, its interactions with βARs will be of interest as it has been suggested that carvedilol could be a prototype for the design of a new generation of therapeutic agents that could stimulate arrestin-dependent signaling, with potentially increased cardioprotective effects, to a greater extent than current beta blockers (Nobles et al., 2011; Noma et al., 2007; Wisler et al., 2007).

The major structural changes that result in binding and activation of a G protein by a 7TMR include large outward movements of transmembrane helices 5 and 6 (Rasmussen et al., 2011b), but the receptor conformational changes that might promote arrestin-mediated signaling or signaling through other G protein-independent pathways in preference to G protein activation are at present unknown. In the case of the β_2_AR, ligands that stimulate arrestin signaling are thought to cause conformational changes at the receptor's C terminus, and these might promote phosphorylation and facilitate interactions with regulatory proteins such as arrestin (Granier et al., 2007). Small increases in phosphorylation levels of the β_2_AR C terminus have been demonstrated for some arrestin-biased agonists, including carvedilol (Drake et al., 2008), and the resulting pattern of phosphorylation is distinct from that promoted in response to stimulation with the nonbiased agonist, isoprenaline (Nobles et al., 2011).

Bucindolol and carvedilol are clearly of interest in relation to their role in the activation of non-G protein-mediated signaling pathways, and, in addition, they are also being studied with respect to two isoforms of human β_1_AR in the treatment of congestive heart failure. A common single nucleotide polymorphism occurs in amphipathic helix 8 (H8) of human β_1_AR, where Arg389^8.56^ is substituted by Gly in 20%–40% of the population depending on ethnicity (Maqbool et al., 1999). The Arg/Gly389 polymorphism results in a significant variation in G protein activation, with β_1_AR-Arg389 having a slightly higher basal activity and a 3-fold increased adenylyl cyclase activity on stimulation with the nonbiased agonist isoprenaline compared to β_1_AR-Gly389 (Mason et al., 1999). Both carvedilol and bucindolol have been shown to be more effective than other beta blockers as inverse agonists of β_1_AR-Arg389 (Liggett et al., 2006; Rochais et al., 2007), findings which were initially heralded as a starting point for personalized therapies in heart failure (DeGeorge and Koch, 2007; Pleger and Koch, 2006).

In order to elucidate the interactions of these ligands with the receptor as a foundation for further studies of signaling with biased agonists, and to determine possible reasons for the enhanced activities of the ligands on β_1_AR-Arg389, we have determined the cocrystal structures of a thermostabilized avian β_1_AR with bucindolol and carvedilol.

## Results and Discussion

### Structures of β_1_AR Bound to Bucindolol and Carvedilol

The avian β_1_AR was modified to allow crystallization by the removal of flexible regions at the N terminus, C terminus, and in cytoplasmic loop 3, and the introduction of 8 point mutations (see Experimental Procedures), to give the construct β_1_AR44-m23. Six of the point mutations result in thermostabilization of β_1_AR (Serrano-Vega et al., 2008), which allows crystallization in short-chain detergents (Warne et al., 2008). A consequence of thermostabilization is that the receptor is preferentially in the antagonist-bound state (R) (Serrano-Vega et al., 2008), although β_1_AR-m23 is capable of binding agonists with a similar rank order of potency to the wild-type receptor and can couple efficiently to G proteins (Baker et al., 2011). It is not feasible to perform crystallography on human β_1_AR due to its extreme instability (Serrano-Vega and Tate, 2009), but the high sequence identity between the receptors (82%) in the transmembrane domains and loop regions (except most of cytoplasmic loop 3) shows β_1_AR-m23 is an excellent model for studying ligand-receptor interactions. Indeed, crystal structures of β_1_AR-m23 (Warne et al., 2008) and β_2_AR-T4L (Cherezov et al., 2007) show high similarity in the transmembrane domains (rmsd 0.7 Å) and especially in the region of the ligand binding pocket (rmsd of 0.25 Å for 78 Cα atoms). None of the mutations in β_1_AR-m23 are in the ligand binding pocket and none of the sites of the mutations show a conformational change when comparing the structures of β_1_AR-m23 and β_2_AR-T4L.

**Figure 2:**
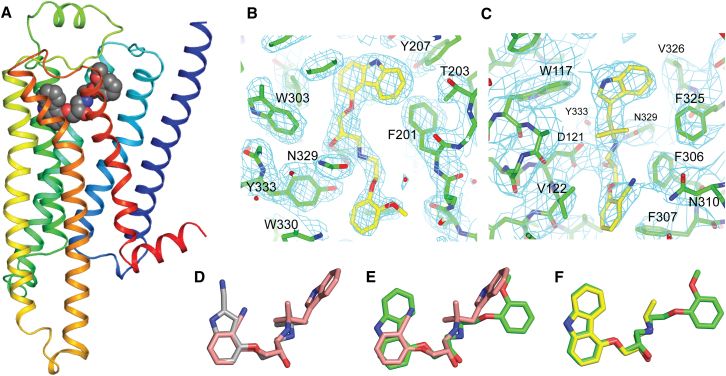
Structure of β_1_-Adrenoceptor bound to Carvedilol (A) The structure of β1AR bound to carvedilol, with the receptor monomer in rainbow coloration with the N terminus in blue and the C terminus in red. Carvedilol is shown in space-filling representation (C, gray; O, red; N, blue). The intracellular side is at the bottom of the figure. (B) Omit map (2Fo-Fc) density for carvedilol (contour level 1.3 σ). (C) Omit map (2Fo-Fc) density for bucindolol (contour level 1.0 σ). (D–F) Alignment of β1AR structures was performed and the superposition of the ligands are depicted. (D) Alignment of bucindolol (pink) and cyanopindolol (gray, PDB code 2VT4). (E) Alignment of bucindolol (pink) with carvedilol (green). (F) Alignment of carvedilol (green) with carazolol (yellow, PDB code 2YCW).

Receptors were expressed, purified and crystallized as previously described. The cocrystal structures of β_1_AR44-m23 bound to either bucindolol or carvedilol were solved at resolutions of 3.2 and 2.3 Å, respectively (Figure 2 and Table 1). Overall the two structures are very similar (RMSD of Cα positions <0.5 Å) to the inactive state structure of β_1_AR (Protein Data Bank [PDB] 2VT4) with bound cyanopindolol (Warne et al., 2008). The lack of any significant conformational change at the ends of helices 5 and 6 is consistent with previous R-state structures of β_1_AR-m23 with bound antagonists and agonists and of β_2_AR complexed with either antagonists or with a covalently bound agonist. Thus, the structures presented here of β_1_AR bound to either bucindolol or carvedilol show in detail the ligand-receptor interactions (see below), but they do not define a new conformation of the receptor involved in G protein-independent signaling. It is likely that a complex of a phosphorylated βAR with arrestin will be required to fully understand the conformational change induced by the binding of biased agonists, because the signaling conformation of the receptor that allows arrestin binding is likely to be transient. This is consistent with the observation that the binding of a G protein or analog was required to obtain the structure of an R^∗^ state of β_2_AR (Rasmussen et al., 2011a, 2011b), while the structure of β_2_AR bound to a covalent agonist is in the R state (Rosenbaum et al., 2011). Therefore, it seems unlikely that any key conformational changes are missing due to the presence of the thermostabilizing mutations. In contrast to the βARs, some receptors evince considerable conformational changes when crystallized in the presence of an agonist. For example, the structure of the adenosine A_2A_ receptor bound to an agonist is clearly in an R^∗^-like state, without the requirement for binding a G protein or G protein mimetic (Lebon et al., 2011; Xu et al., 2011).

### Ligand Binding in the Catecholamine Binding Pocket

**Figure 3:**
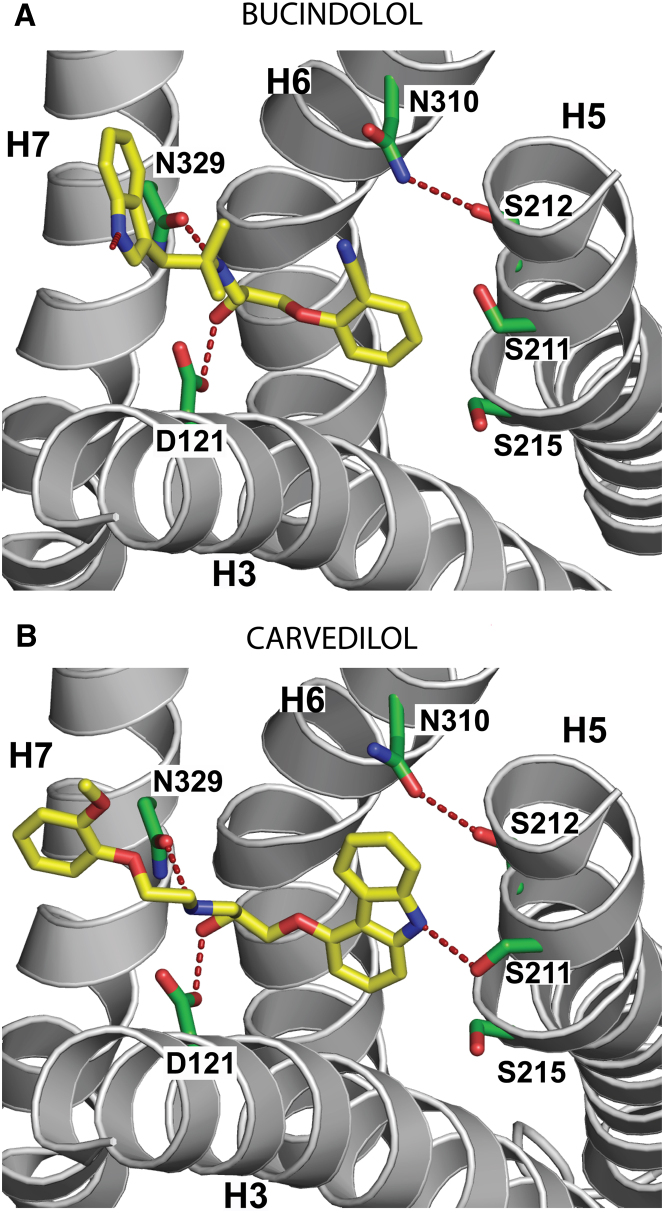
Structures of the Ligand Binding Pocket β_1_AR-m23 is depicted as a cartoon (gray) viewed from the extracellular surface with specific side chains (C, green; N, red; O, blue) depicted making potential hydrogen bonds (red dashed lines) to the ligand, as well as the interhelical potential hydrogen bond between Ser212^5.43^ and Asn310^6.55^. The ligand coloring scheme is C, yellow; N, red; O, blue. (A) Bucindolol. (B) Carvedilol. Note the alternative rotamer conformation of Asn310^6.55^ depicted in the carvedilol-bound structure in which the amide oxygen faces toward H5. In this configuration a potential steric clash with the ligand is avoided, this conformation could be dependent on the nature of the ligand.

The structures of βAR ligands are often very similar, particularly in the region of the secondary amine and β-hydroxyl groups and these are also conserved in bucindolol and carvedilol (Figure 1). The cocrystal structures show that their secondary amine and β-hydroxyl groups form potential hydrogen bonds with Asp121^3.32^ and Asn329^7.39^ and their “head groups” (equivalent to the catechol moiety in adrenaline) occupy a position adjacent to H5 (Figure 3). Thus, both ligands exhibit the same general mode of binding observed for other βAR antagonists cocrystallized with either β_1_AR or β_2_AR (Cherezov et al., 2007; Hanson et al., 2008; Moukhametzianov et al., 2011; Wacker et al., 2010; Warne et al., 2008).

**Figure 4:**
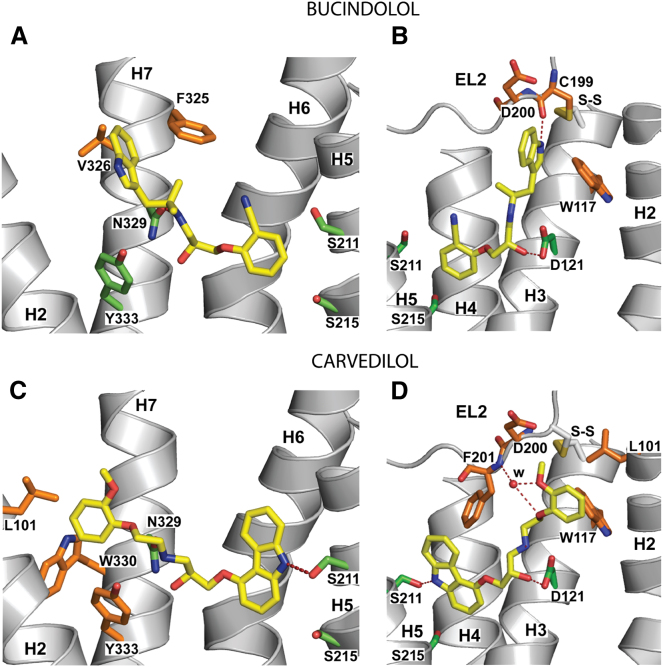
Key Receptor-Ligand Interactions between Bucindolol, Carvedilol, and β_1_AR Receptor structures are shown in cartoon representation as viewed in the membrane plane with the three helices obscuring the binding site removed for clarity and the remaining helices labeled: (A and B) monomer A of the bucindolol complex; (C and D) monomer B of the carvedilol complex. Amino acid residues within 3.9 Å of the ligands (yellow) are depicted in stick representation; green, residues with previously known ligand interactions; orange, residues in the extended ligand binding pocket interacting with either the indole or methoxyphenoxy substituents on the ligands. Atoms are colored accordingly; C, yellow, green, orange; O, red; N, blue. Potential hydrogen bonds are shown as red dashes. Carvedilol makes a polar contact with EL2 mediated by a bridging water molecule (red sphere labeled w; B-factor 42 Å^2^). The disulphide bond (labeled S-S) between Cys199 on EL2 and Cys114^3.25^ on H3 is shown in (B) and (D). For a full list of receptor-ligand interactions and Ballesteros-Weinstein nomenclature, see Table 2.

Previously, we showed that binding of a full agonist to β_1_AR resulted in three differences in receptor conformation compared to when an antagonist was bound, namely the rotamer conformation changes of Ser212^5.43^ and Ser215^5.46^, and a contraction of the ligand binding pocket. Comparison of the structures of β_1_AR bound to bucindolol and carvedilol with the previously determined structures shows that the rotamer configuration of Ser215^5.46^ allows the formation of an interhelical hydrogen bond with H3, as seen in the structures with bound partial agonists, but not with bound full agonists where Ser215^5.46^ forms a hydrogen bond directly to the ligand (Figure 4). However, the configuration of Ser212^5.43^ is similar to that seen in the agonist-bound structures where it makes a potential hydrogen bond to Asn310^6.55^. A recently determined structure of β_1_AR with cyanopindolol bound in lipidic meso phase at a resolution of 2.1 Å (J.L. Miller and C.G.T., unpublished data) also shows this alternative rotamer conformation of Ser212^5.43^ compared to the previous structure of cyanopindolol-bound β_1_AR determined in detergent (Warne et al., 2008); these data suggest that Ser212^5.43^ can also be in this alternative conformation with an antagonist bound, and the configuration of Ser212^5.43^ is therefore unlikely to represent an agonist-specific conformation. Both the bucindolol and carvedilol bound structures also do not exhibit the contraction of the binding pocket observed in the structures with full and partial agonists bound. It is therefore clear that the ligands do not induce the initial conformational changes in the receptor that are characteristic of agonists that activate G proteins. Bucindolol has been variously reported as being either an inverse agonist or partial agonist of the β_1_AR, depending on the system or tissue studied (Andreka et al., 2002; Engelhardt et al., 2001; Galandrin et al., 2008; Maack et al., 2000, 2003). However, in both bucindolol-bound and carvedilol-bound β_1_AR structures, the characteristic rotamer conformation change of Ser215^5.46^ observed in structures with a full agonist bound is sterically blocked by the ligand, which is a characteristic of βAR inverse agonists (Warne et al., 2011).

### Ligand Binding in the Extended Ligand Binding Pocket

Both bucindolol and carvedilol, unlike all other antagonists cocrystallized with βARs, have bulky aromatic substituents at their amine ends that make additional contacts in the extended ligand binding pocket composed of residues in helices 2, 3, and 7 and extracellular loop 2 (Figure 4). The additional contacts are detailed along with all other ligand-receptor contacts in Table 2. It therefore seems logical to propose that the ability of bucindolol and carvedilol to stimulate G protein-independent signaling resides in the extensions in the tail region of the ligand that are absent from all other antagonists. However, it is difficult to say how these additional contacts might promote G protein-independent signaling, because the conformations that might finally promote the binding of arrestin or other signaling proteins are currently unknown. However, it seems plausible that the additional contacts may result in an increased probability of subtle conformational changes that might be transmitted to the receptor's C terminus, where phosphorylation by GPCR-specific kinases promotes binding of arrestin and signaling (Granier et al., 2007; Nobles et al., 2011).

Bias of conventional G protein agonists of βARs toward the arrestin-signaling pathway has been investigated and methods to discern levels of G protein-independent activity that are relatively low compared to the dominant G protein-signaling activity are being developed. This has led to the categorization of a number of ligands that can activate both G protein-coupled and G protein-independent pathways, but to different extents (Rajagopal et al., 2011). However, currently there is not a comprehensive list of the propensity of all βAR ligands for inducing signaling via G protein-independent pathways. So far, bias toward arrestin signaling among conventional G protein agonists has only been detected in ligands with either ethyl substitutions at the Cα, or amine-end substituents, such as those present in bucindolol and carvedilol (Drake et al., 2008; Rajagopal et al., 2011). Of the conventional G protein agonists that have been cocrystallized previously with either β_1_AR or β_2_AR, dobutamine, isoprenaline and salbutamol have been identified as nonbiased agonists of the β_2_AR (Rajagopal et al., 2011). The structure of β_1_AR bound to carmoterol has been determined (Warne et al., 2011), but this ligand has not been tested for signaling bias. However, formoterol, which is weakly arrestin biased, is structurally identical to carmoterol apart from a minor difference in its head group; both ligands have the same methoxyphenyl amine end extension (see Figure 1 for the structures) (Rajagopal et al., 2010). The cocrystal structure of carmoterol with the β_1_AR indicates additional interactions of the methoxyphenyl group with extracellular loop 2 (EL2) as well as H7, and NMR data suggest that formoterol's methoxyphenyl group also interacts with residues on EL2 of the β_2_AR (Bokoch et al., 2010). Thus, of the three ligands with amine-end extensions that bind in the extended ligand binding pocket for which there are cocrystal structures and that have been examined for bias, bucindolol and carvedilol are biased agonists, whereas dobutamine is not. As in all probability, formoterol, which also shows weak arrestin bias also binds to EL2, one structural feature that may correlate with agonist bias is that bucindolol, carvedilol, and most likely formoterol all interact with EL2, whereas dobutamine does not. Whether this observation extends to other biased agonists will require further detailed characterization of more βAR ligands with both β_1_AR and β_2_AR rather than just a limited few.

While this manuscript was in review, a related manuscript appeared (Liu et al., 2012), which detailed ^19^F-NMR studies on β_2_AR bound to various ligands. Specific Cys residues in detergent-solubilized, purified β_2_AR were covalently modified with trifluoroethanethiol and then ^19^F spectra were collected in the presence of inverse agonists, agonists, or biased agonists. The most significant difference observed when an inverse agonist was bound compared to when a biased agonist was bound was a change in the spectrum of ^19^F-labeled Cys327; this residue is in the short linker between H7 and H8. These data indicate that the environment around Cys327 is different when a biased agonist is bound compared to when an inverse agonist is bound, which is consistent with the interpretation of the structural data presented here and previous biochemical data (Granier et al., 2007; Nobles et al., 2011).

### Understanding the Effects of the Arg389Gly Polymorphism in Human β_1_AR

The high-resolution of the β_1_AR-carvedilol complex fortuitously also allows us to suggest a mechanism for the difference in pharmacology in the frequently occurring Arg389Gly polymorphism of the human β_1_AR. The more common β_1_AR-Arg389 variant has a slightly higher basal G protein activity and a 3-fold increase in agonist response compared to the β_1_AR-Gly389 variant (Mason et al., 1999). It has been suggested that the affected residue is in an area important for G protein coupling (Mason et al., 1999), but it is now clear from the structure of the β_2_AR-Gs complex that this is not the case (Rasmussen et al., 2011b). Therefore, the differences in pharmacology between the Arg/Gly variants must lie within the receptor itself.

**Figure 5:**
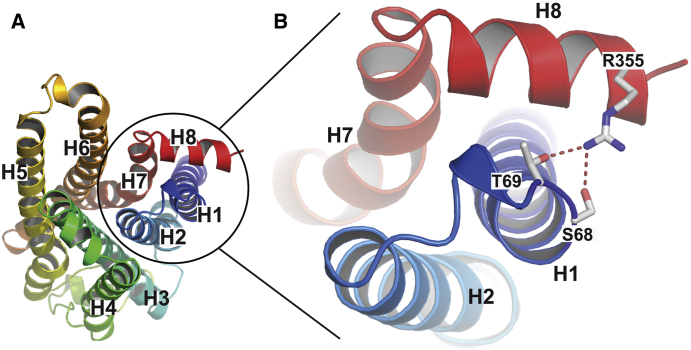
Interactions between Amphipathic H8 and H1 (A) The structure of carvedilol-bound β_1_AR-m23 is shown as viewed from the cytoplasmic surface in rainbow coloration (N terminus blue, C terminus red) and the helices labeled accordingly (H1-H8). (B) Region depicting the potential hydrogen bonds (red dashed lines) between side chains in H1 and H8. The electron density for Arg355 suggests that alternative rotamer conformations are present, but the conformation depicted represents the most favored.

The human β_1_AR residue affected by the polymorphism, Arg389^8.56^, is equivalent to Arg355^8.56^ in the turkey β_1_AR and it is located on H8, with its guanidinium group close to the end of H7. In the 2.3 Å resolution structure of carvedilol-bound β_1_AR-m23, the side chain of Arg355^8.56^ is well resolved for the first time in any structure (Figure 5) and it forms hydrogen bonds with Ser68^1.59^ and Thr69^1.60^ at the intracellular end of H1. The hydrogen bonds between H8 and H1 would be expected to stabilize the receptor, which is substantiated by the fact that Ser68^1.59^ is one of the six thermostabilizing mutations that facilitated crystallization of β_1_AR-m23 (Serrano-Vega et al., 2008; Serrano-Vega and Tate, 2009). In the wild-type turkey β_1_AR, the equivalent residue to Ser68^1.59^ is Arg68^1.59^ and when this is mutated to an uncharged residue (Ala) the receptor becomes more thermostable, presumably because the proximity of Arg68^1.59^ and Arg355^8.56^ is electrostatically unfavorable. Mutation of Arg68^1.59^ to Ser increases the thermostability further, which is consistent with the formation of the hydrogen bond to Arg355^8.56^ observed in the structure with bound carvedilol. Increased thermostability of β_1_AR is likely to reflect a decrease in the global flexibility of the whole receptor. Thus, changes in thermostability due to mutations at the H1-H8 interface suggests that analogous changes in homologous receptors may also change the global dynamics of these receptors. These observations in the avian β_1_AR probably apply to human β_1_AR and β_2_AR as the equivalent residues to Arg68^1.59^ are Lys85^1.59^ and Lys60^1.59^. Thus, the Arg/Gly389 polymorphism in human β_1_AR will similarly alter the packing between H1 and H8, which could result in changes in the dynamics of the receptor, with the barrier to formation of the R^∗^ state perhaps being lower in the β_1_AR-Arg389 variant. This is indeed what has been observed pharmacologically and biochemically in a number of studies (Liggett et al., 2006; Rochais et al., 2007; Swift et al., 2008). It has also been observed that carvedilol is a more efficient inverse agonist of the human β_1_AR-Arg389 isoform than are metoprolol and bisoprolol (Rochais et al., 2007), two beta blockers that do not have extensions at their amine ends (see Figure 1). These findings have led to interest in the potential of genetically targeted therapies for heart failure with bucindolol or carvedilol (DeGeorge and Koch, 2007; Pleger and Koch, 2006). A similar polymorphism at Trp64^1.59^ in the human β_3_AR has also been observed, where substitution of Trp for Arg has been associated with compromised activity of the receptor and, consequently, increased obesity (Kimura et al., 2000; Kurokawa et al., 2008; Piétri-Rouxel et al., 1997).

### Conclusions

The structures of β_1_AR bound to either bucindolol or carvedilol show that both of these ligands make additional contacts to helices 2, 3, and 7 and extracellular loop 2 compared to other structurally characterized βAR inverse agonists. Overall the structures show no conformational change when compared to other β_1_AR antagonist structures, but it is probable that the additional interactions in the extended ligand binding pocket might increase the likelihood of subtle conformational changes that result in enhanced arrestin binding and G protein-independent signaling. The fact that both bucindolol and carvedilol bind to β_1_AR in a similar manner to other βAR G protein antagonists, yet they can stimulate signaling via G protein-independent pathways while apparently inhibiting G protein coupling, strongly supports the contention that arrestin can bind to a different conformation of the receptor to that bound by G proteins.

## Experimental Procedures

### Expression, Purification, and Crystallization

The turkey (*Meleagris gallopavo*) β_1_AR construct, β44-m23, contains six thermostabilizing point mutations, two point mutations which improve receptor expression and homogeneity, and truncations at the N terminus, inner loop 3 and C terminus (Warne et al., 2011). Baculovirus expression and purification were all performed as described previously, with the detergent exchanged to Hega-10 (0.35%) on the alprenolol Sepharose affinity column (Warne et al., 2003, 2008, 2009, 2011). Purified receptor was competitively eluted from the alprenolol Sepharose column with either bucindolol or carvedilol, but this was difficult because of the poor solubility of the ligands. Ligands were added to saturation from 20 mg/ml DMSO stock solutions to the elution buffer (10 mM Tris-HCl [pH 7.4], 100 mM NaCl, 0.1 mM EDTA, 0.35% Hega-10) with rapid stirring. The approximate final concentrations of the ligands in the elution buffer were 10 μM for carvedilol and 50 μM for bucindolol. Receptor was concentrated to 20 mg/ml in 10 mM Tris-HCl (pH 7.6), 100 mM NaCl, 0.1 mM EDTA, 0.35% Hega-10. Before crystallization, Hega-10 was added to 0.5%. Crystals were grown at 4°C by vapor diffusion in sitting drops with 150 nl receptor + 150 nl precipitant (0.1 M bicine [pH 9.0], 25% PEG 600 in both cases) and cryoprotected by addition of 60% PEG 600 for 1 min before mounting on Hampton CrystalCap HT loops and cryocooling in liquid nitrogen.

### Data Collection, Structure Solution, and Refinement

For both complexes, diffraction data were collected from a single cryocooled crystal (100 K) at the European Synchrotron Radiation Facility, Grenoble, France, with a Mar 225 CCD detector on beamline ID23-2 (wavelength, 0.8726 Å) using a 10 μm focused beam. The microfocus beam was required for the location of the best diffracting parts of crystals, as well as allowing wedges of data (20–80°) to be collected from different positions on the crystal. For the β_1_AR crystals grown in the presence of bucindolol or carvedilol, 9 or 16 wedges of data from single crystals were merged, respectively. Images were processed with MOSFLM (Leslie, 2006) and SCALA (Evans, 2006). Both structures were solved by molecular replacement with PHASER using the β_1_AR44-m23 structure with the agonist carmoterol bound (PDB code 2Y02) as a starting model (McCoy et al., 2007). Refinement, rebuilding and validation were carried out with REFMAC5 (Murshudov et al., 1997), COOT (Emsley and Cowtan, 2004) and MOLPROBITY (Davis et al., 2007). Noncrystallographic symmetry restraints were applied as appropriate between the two monomers in the asymmetric unit for both structures, using the electron density maps and R_free_ values to judge which residues should be excluded. The two independent copies of the receptor in the asymmetric unit are very similar for the bucindolol complex, although the ligand density was better defined for monomer A. In the carvedilol complex, there is a distortion of the ligand binding pocket in monomer A due to lattice contacts and monomer B represents the more physiologically relevant conformation.

## Figures and Tables

**Table 1:** Data Processing, Refinement, and Evaluation Statistics

	β44-m23 + Bucindolol	β44-m23 + Carvedilol
Space group	P2_1_	P2_1_
Cell dimensions *a, b, c* (Å), β (^o^)	89.8, 60.7, 107.8, 110.8	90.1, 62.2, 100.9, 109.2

**Data Processing**		

Resolution (Å)	52.1–3.2	52.1–2.3
Rmerge^a^	0.102 (0.596)	0.099 (0.593)
<I/σ(I)>^a^	9.9 (2.5)	11.3 (2.1)
Completeness (%)^a^	95.9 (86.5)	99.8 (99.1)
Multiplicity^a^	5.3 (5.4)	6.2 (4.0)
Wilson B factor (Å^2^)	103.6	37.5

**Refinement**		

Total number of reflections	16,557	44,731
Total number of atoms	4,673	4,962
Number of waters	13	100
Number of detergent molecules	5	12
Number of sodium ions	0	2
R_work_b,c	0.242 (0.350)	0.202 (0.288)
R_free_c,d	0.279 (0.384)	0.240 (0.315)
Rmsd bonds (Å)	0.005	0.012
Rmsd angles (^o^)	0.984	1.39
Mean atomic B factor (Å^2^)	92.7	39.2
Estimated coordinate error (Å)	0.35	0.125
Ramachandran plot favored (%)^e^	98.2	98.1
Ramachandran plot outliers (%)^e^	0	0

a Values in parentheses are for the highest resolution bin (Å) (bucindolol, 3.37–3.2; carvedilol, 2.42–2.30).

b Number of reflections used to calculate R_work_ (bucindolol, 15,659 [94.9%]; carvedilol, 42,352 [95.0%]).

c Values in parentheses are for the highest resolution bin for refinement (Å) (bucindolol, 3.28–3.20; carvedilol, 2.36–2.30).

d Number of reflections from a randomly selected subset used to calculate R_free_ (bucindolol, 898 [5.1%]; carvedilol 2,378 [5.0%]).

e Figures obtained using MolProbity (Davis et al., 2007).

**Table 2:** Amino Acid Side-Chain Contacts between β_1_AR and Ligands

Amino Acid Residue	B-W Number	Secondary Structure	Bucindolol Monomer A	Carvedilol Monomer B	Carazolol 2YCW Monomer A	Cyanopindolol 2VT4 Monomer B
Leu101	2.64	H2	—	v der W	—	—
Trp117	3.28	H3	v der W	v der W	v der W	v der W
Thr118	3.29	H3	v der W	—	—	v der W
Asp121	3.32	H3	H-bond	H-bond	H-bond	H-bond
Val122	3.33	H3	v der W	v der W	v der W	v der W
Val125	3.36	H3	v der W	v der W	v der W	—
Cys199	—	EL2	H-bond	—	—	—
Asp200	—	EL2	v der W	v der W	—	—
Phe201	—	EL2	v der W	v der W (H-bond via water)	v der W	v der W
Thr203	—	EL2	—	—	—	Polar
Tyr207	5.38	H5	—	v der W	v der W	—
Ala208	5.39	H5	—	—	—	v der W
Ser211	5.42	H5	v der W	H-bond	H-bond	H-bond
Ser215	5.46	H5	v der W	v der W	v der W	v der W
Trp303	6.48	H6	v der W	v der W	v der W	v der W
Phe306	6.51	H6	v der W	v der W	v der W	v der W
Phe307	6.52	H6	v der W	v der W	v der W	v der W
Asn310	6.55	H6	v der W	v der W	v der W	H-bond
Phe325	7.35	H7	v der W	—	—	—
Val326	7.36	H7	v der W	—	—	—
Asn329	7.39	H7	H-bond	H-bond	H-bond	H-bond
Trp330	7.40	H7	—	v der W	—	—
Tyr333	7.43	H7	v der W	v der W	v der W	v der W

## Box 1 Pharmacological Definitions

**Agonist.** A ligand that binds to and activates a receptor and elicits a physiological response. The endogenous agonist for the β_1_AR is noradrenaline, which is a full agonist that elicits the maximal response for the receptor in activating a G protein.

**Constitutive or basal activity.** Physiological response that occurs in the absence of any receptor-activating ligand due to a fraction of the receptor being in an activating conformation.

**Inverse agonist.** A ligand that binds to a receptor and inhibits or eliminates, in the case of a full inverse agonist, the basal or constitutive activity of a receptor.

**Partial agonist or partial inverse agonist.** Ligands that elicit only a partial response when compared to either a full agonist or a full inverse agonist, respectively.

**Antagonist.** Any ligand that blocks binding of endogenous agonists to the receptor, thereby modulating receptor activity. A general term that encompasses ligands that may be inverse agonists, partial agonists, or neutral in effect.

**Beta blockers.** Term for antagonists of βARs when used in a therapeutic setting.

**Biased agonist.** A ligand that binds to a receptor and signals to a variable extent through both G protein-dependent and G protein-independent pathways. A ligand may be “weakly biased,” i.e., signaling is mainly via G proteins or “perfectly biased,” i.e., the ligand is an inverse agonist of the G protein-coupled pathway as well as an agonist of the G protein-independent pathways.

**Note.** The categorization of ligands can be dependent on the type of assay used, the cell type in which the receptor has been expressed, expression levels, and the sensitivity and dynamic range of the assay used to detect downstream signaling events.

## Accession Numbers

Coordinates and structure factors have been submitted to the PDB database under accession codes 4AMI and 4AMJ for b44-m23 bound to either bucindolol or carvedilol, respectively.
